# Multifunctional injectable hydrogel for effective promotion of cartilage regeneration and protection against osteoarthritis: combined chondroinductive, antioxidative and anti-inﬂammatory strategy

**DOI:** 10.1080/14686996.2022.2076568

**Published:** 2022-06-01

**Authors:** Xueping Dong, Canfeng Li, Mengdi Zhang, YiKun Zhao, Zhen Zhao, Wenqiang Li, Xintao Zhang

**Affiliations:** a Department of Sports Medicine and Rehabilitation, Peking University Shenzhen Hospital, Shenzhen, PR China; b School of Clinical Medicine, Weifang Medical University, Weifang, PR China; c Engineering Technology Research Center for Sports Assistive Devices of Guangdong, Guangzhou Sport University, Guangzhou, PR China

**Keywords:** Dual-crosslinking hydrogel, chondrogenic differentiation, anti-inflammation, cartilage regeneration, OA

## Abstract

The regeneration of the articular cartilage defects is characterized by the improvement in the quality of the repaired tissue and the reduction in the potential development of perifocal osteoarthritis (OA). Usually, the injection of dexamethasone (Dex) in the OA joints slows down the progression of inflammation and relieves pain. However, the anti-inflammatory Dex injected in the joint cavity is rapidly cleared, leading to a poor therapeutic effect. Multifunctional hydrogels with simultaneous chondrogenic differentiation, antioxidative, and anti-inﬂammatory capacities may represent a promising solution. Therefore, in this work, a novel injectable hydrogel based on double cross-linking of Schiff base bonds and coordination of catechol-Fe was developed. The obtained hydrogel (Gel-DA/DOHA/DMON@Dex@Fe) possessed molding performance in situ, excellent mechanical strength, controllable biodegradability, the on-demand release of the drug, and biocompatibility. The hydrogel system stimulated the HIF-1α signaling pathway and suppressed inflammation thanks to the introduction of DMON@Fe, consequently facilitating chondrogenic differentiation. The synergistic anti-inflammatory effect together with the induction of chondrogenesis by Dex-loaded Gel-DA/DOHA/DMON@Fe hydrogel allowed the promotion of cartilage repair, as demonstrated by *in vivo* experiments. Hence, the proposed multifunctional scaffold provides a promising advancement in articular cartilage tissue engineering and may have great prospects in the prevention of OA.

## GRAPHICAL ABSTRACT

**Figure:**
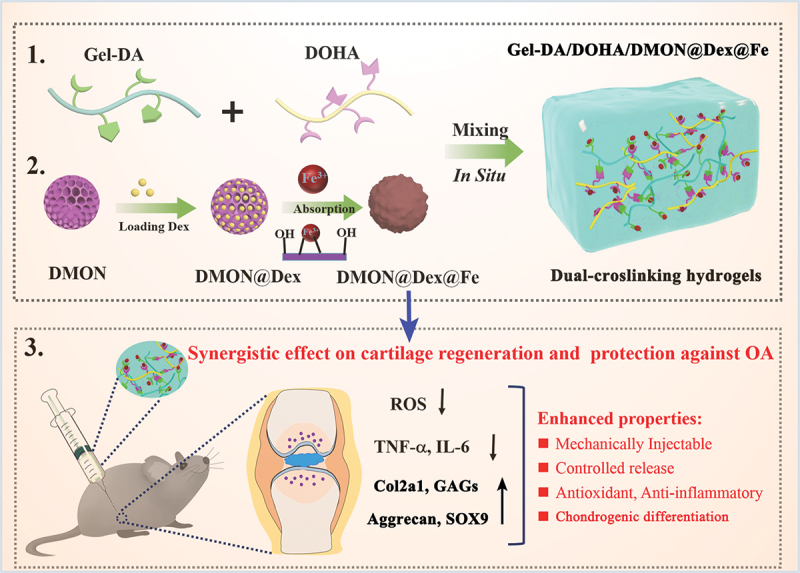

## 1. Introduction

The self-repair ability of the articular cartilage after the injury is limited due to the lack of vascular, neural, and lymphatic systems. Therefore, articular cartilage defects have become a serious clinical concern, since the development of osteoarthritis (OA) may occur in such cartilage defects, further causing joint dysfunction [1–3]. At present, hydrogel-based tissue engineering represents a promising strategy for cartilage defects. Indeed, this approach has been extensively investigated because hydrogels can be designed with a particular scaffold that promotes cell proliferation and the production of cartilage-specific extracellular matrix (ECM) [4,5]. The catechol-chemistry-based hydrogels are characterized by long-term adhesiveness, excellent antibacterial properties, and antioxidant performance, leading to the development of multifunctional composite hydrogel scaffolds usable in tissue engineering [6,7]. For example, a hydrogel based on catechol-Fe coordinate dynamic bond was developed with scavenging ability of reactive oxygen species (ROS) [8]. In addition, a multifunctional hydrogel with hyaluronic acid-graft-dopamine internally doped with reduced graphene oxide was reported as inducing tissue regeneration [9]. However, the above-mentioned hydrogels cannot guarantee an appropriate injectability because of commonly mechanically.

The restoration of cartilage diseases usually relies on joint replacement by surgery (i.e. arthroplasty), which is often followed by huge risks of severe pain and inflammation, significantly affecting the process of articular cartilage repair and regeneration [10,11]. Under normal circumstances, the injection of the anti-inflammatory drug dexamethasone (Dex) in OA joints and other inflamed tissues slows down the progression of inflammation and relieves the pain [12]. However, Dex injected in the joint cavity is rapidly cleared because of unsatisfactory biocompatibility, leading to a poor therapeutic effect. Thus, it is of utmost importance the development of a new approach to repair cartilage guaranteeing the release of Dex at the site of inflammation and on-demand release of the drug in the specific location where the lesions are present to improve the healing of cartilage defects. The dendritic mesoporous organic silica nanoparticles (DMON) are a promising system for the delivery of various cargos due to the well-defined mesoporous structure, mechanical and chemical stability, excellent biocompatibility, and molecularly biodegradable organic-inorganic hybrid composition [13,14]. In particular, silica-based nanoparticles are able to stimulate chondrogenic differentiation and improve osteogenic activity [15–17]. Another challenge for cartilage regeneration is the overexpression of ROS since it is considered as a mediator of inﬂammation to regulate chondrocyte apoptosis, resulting in tissue injury [18,19]. Many previous studies confirmed that both Si ions and the expression of hypoxia-inducing factor 1α (HIF-1α) are upregulated by Fe^2+^, thus promoting the activation of downstream chondrogenic genes (SOX9, Col2a1, etc.), and switching metabolism from glucose oxidation to glycolysis for satisfying energy requirements that regulate the proliferation and survival of chondrocytes [20,21]. Therefore, an alternative approach is highly necessary for the regulation of the local microenvironment to facilitate the intrinsic chondral regeneration by the effective activation of the HIF-1α signaling pathway.

Considering the above concerns, a specific type of physicochemical dual crosslinking multifunctional hydrogel was designed for cartilage regeneration. In this study, the main-chain segments of the hydrogel were catechol-modified gelatin (Gel-DA) and dopamine-modified oxidized hyaluronic acid (DOHA), and DMON were used to encapsulate Dex by the deposition of a Fe^3+^ layer to achieve a controlled release of the drugs, thus solving the incompatibility of hydrophobic agents in the hydrogel systems by improving the solubility. Overall, the novel Gel-DA/DOHA/DMON@Fe hydrogels loaded with Dex were obtained based on combining Schiff base (amino groups and aldehyde groups) with coordination band (catechol groups and Fe^3+^). The resulting hydrogel system exhibited a reinforced mechanical behavior, good injectable properties, cytocompatibility, as well as antioxidant and anti-inflammatory ability. In addition, a synergistic anti-inflammatory effect and chondroinduction of the cartilage due to the activation of HIF-1α signaling was demonstrated by the *in vitro* and *in vivo* evaluation of hydrogel scaffolds. The above results indicated that the multifunctional Gel-DA/DOHA/DMON@Dex@Fe hydrogels were ideal to repair cartilage defects and to prevent OA, revealing promising prospects for further biomedical applications.

## 2. Experimental section

### 2.1. Materials

The modified Gel-DA (with a degree of substitution of dopamine of 13.6%) and DOHA (The TNBS method was 33.2% measured by oxidation degree of OHA) were supplied by the Biomaterials Research Group at Jinan University. Gelatin (type A, 300 bloom), HA (The *M*_n_ is 150 000– 300,000 Da), Dopamine (DA) hydrochloride, hexadecyl trimethyl ammonium bromide (CTAB), bis[3-(triethoxysilyl)propyl] tetrasulfide (BTES), Tetraethoxysilane (TEOS), and Iron (III) chloride hexahydrate (FeCl_3_•6 H_2_O) were purchased from Sigma-Aldrich. Dexamethasone sodium phosphate (Dex-p) was purchased from Aladdin Biochemical Technology Co., Ltd. (Shanghai, China). Tianjin Damao Chemical Reagent Factory provides the other chemical reagents used in this research.

### 2.2. Synthesis of DMON and DMON@Fe nanoparticles

DMON was synthesized according to the protocol previously reported [22]. Briefly, TEA (0.1 g) was diluted in pure water (20 mL) at 80°C under magnetic stirring for 0.5 h, followed by the addition of NaSal (120 mg) and CTAB (350 mg) to the reaction solution, which was kept under stirring for another 1 h. After that, BTES (3.2 mL) and TEOS (4 mL) were added into a premixed solution, which was added into the above solution, and the whole mix was subjected to a gentle stirring (300 rpm) for another 12 h. The products collected by high-speed centrifugation were washed with water and ethanol three times. Next, the collected products were re-dispersed three times in 1% NaCl methanol solution at room temperature for 6 h each time to remove the template. Then, the DMON (30 mg) obtained above was re-dispersed in deionized water (30 mL) and 200 mg PVP was added under vigorous stirring. Subsequently, the FeCl_3_ solution (150 mM) was added dropwise into the reaction solution under ultrasonic vibration. Finally, the product DMON@Fe nanoparticles were obtained by centrifugation and washing after stirring overnight.

The morphology and distribution of the DMON@Fe nanoparticles were evaluated using field emission scanning electron microscope (FE-SEM, ULTRA 55, Carl Zeiss, Germany) and transmission electron microscopy (TEM, Philips CM-120, Eindhoven, Netherlands). Also, the corresponding elemental mappings and energy-dispersive X-ray spectroscopy (EDS) spectra of products were acquired from TEM. Their surface area and pore size were measured on a Micromeritics Tristar 3000 system by an N_2_ adsorption-desorption isotherm.

### 2.3. Formation of Gel-DA/DOHA/DMON@Fe hydrogels

The Gel-DA with the DOHA were dissolved in deionized water and mixed in a molar ratio of 1:1 with -CHO/-NH_2_. Then, DMON@Fe nanoparticles at a different mass ratio of 0.5, 1.0, and 1.5 wt% (w/v) were added to the above solution. After stirring for 30 min, NaOH (4 M) was used to adjust the pH above 9. Then, the pregel solution was incubated at 37°C for 5 min to obtain the transformation from a nano-particle suspension to hydrogel through the Schiff Base reaction between aldehyde groups and amino groups as well as the coordination cross-linking between catechol groups and DMON@Fe. The tube inverted test was used to evaluate the gelation of Gel-DA/DOHA/DMON@Fe. In addition, the pure Gel-DA/DOHA hydrogel was produced and used for comparison. At last, the PBS solution was used to wash several times and purify the resultant hydrogel samples. The structure of the prepared hydrogels was analyzed by Fourier transform infrared (FTIR) spectroscopy (Fts6000, Bio-Rad, USA) using an attenuated total reflectance (ATR) mode.

### 2.4. Characterization of hydrogels

#### 2.4.1. Rheological measurements

The rheological properties of the hydrogels considered in this study were measured on a rotational rheometer (DHR, TA Instruments, USA) at 37°C. Firstly, the gelation time of the mixtures was determined through time sweeps at a frequency of 6.28 rad/s and oscillatory strain of 1%. Then, frequency sweep oscillatory tests were carried out at a constant strain of 1%, with amplitude frequencies ranging from 0.1 to 100 rad/s. Finally, the strain sweep was carried out to confirm that the value of this modulus was within the linear elastic regime (oscillatory strain from 0.1% to 100% and frequency of 6.28 rad/s).

#### 2.4.2. Mechanical behavior

The compression tests were carried out on a mechanical testing machine (AGI-1, Shimadzu, 1 kN load, Japan) to evaluate the prepared hydrogel samples that were cylindrical (8 mm thickness × 10 mm diameter) at room temperature. Furthermore, cycling compression tests were performed to measure the properties of compression and recovery from 0% to 70% and at a speed of 2 mm/min, and the cycle was repeated 20 times. As regards the tensile test, the dumbbell-shaped samples were fixed between two pieces of double-sided tape and then extend at a rate of 1 mm/min by a mechanical testing machine (AGI-1, Shimaodzu, 1 kN load, Japan until failure).

#### 2.4.3. SEM observation

The resultant hydrogel scaffolds were transferred to freezer with −80°C and dried by freeze dryer. Then the freeze-dried specimen were cryogenically fractured under liquid nitrogen condition, and treated by gold spraying equipment (Sputter Coater, Ted Pella, LJ-16) for SEM observation, and the pore diameter was analyzed by Image J Software.

#### 2.4.4. Swelling property

The swelling ratio was measured according to a previously reported approach [23]. The resulting Gel-DA/DOHA/DMON@Fe hydrogel with different amounts of DMON@Fe nanoparticles was fully immersed in a PBS solution at pH 7.4 (n = 3) for 24 h to obtain a complete swelling.

### 2.4.5. In vitro degradation performance

The initial weight of the resulting Gel-DA/DOHA and Gel-DA/DOHA/DMON@Fe was recorded (*W*_0_). Then, the hydrogels were added into a PBS solution. After soaking, the hydrogels were collected, rinsed with water, lyophilized, and weighed again (*W*_T_) at intervals. The study lasted 40 days in a shaker at 37°C. The reduced weight value was calculated as follows:

(W0−WT)/W0×100%

### 2.5. Dex loading and drug release in vitro

Dex was encapsulated into DMON@Fe nanoparticles prior to forming a gel. Briefly, DMON (10 mg in 10 mL solution) was mixed with 1 mg Dex (dissolved in 0.5 mL DMSO) under sonication. Then, the Fe^3+^ layer was placed on the surface of DMON by electrostatic adsorption as described in the above method described in Section 2.2.2. The formed DMON@Dex@Fe was washed three times with pure water using Amicon tubes (molecular weight cut-off, 10 kDa; Millipore) to remove the free drug. The supernatant and washing solution were used to measure the loading amount by UV-Vis at a wavelength of 242 nm. Subsequently, the loaded-Dex Gel-DA/DOHA/DMON@Fe hydrogels were prepared using the same as described above in Section 2.3.3. Also, the prepared Dex-loaded Gel-DA/DOHA/DMON@Fe hydrogels were washed three times with PBS to remove the reaction residue on the surface of the hydrogel and collected these washing solution for determining the amounts of unloaded Dex of Gel-DA/DOHA/DMON@Dex@Fe by a UV–vis spectrophotometer. The loading efficiency was the mass percentage of the loaded Dex to the feeding Dex.

As regards the drug release analysis, the resulting Gel-DA/DOHA/DMON@Dex@Fe samples were examined in 0.1 M PBS at pH 7.0 and pH 6.5. The Dex-loaded hydrogel was placed in 0.2 mL PBS and shaken at 100 rpm and 37°C. The supernatant was replaced and corrected with an equal amount of fresh solution within a predetermined time interval. The amount of the released Dex was measured by UV absorbance at 242 nm and the release profile was obtained.

### 2.6. In vitro cell experiment

#### 2.6.1. Cytocompatibility evaluation

Bone marrow mesenchymal stem cells (BMSCs) were used to study the cytocompatibility of the hydrogel system. After sterilization, the hydrogel scaffolds (10 mm in diameter and 1 mm in thickness) were immersed in the culture medium for 2 h. Then, the cells were seeded on the surface of the hydrogel scaffold at a density of 2 × 10^4^ cells. The medium was removed and replaced every 2 days. Cells used as a control group were seeded in a culture plate at the same density of 2 × 10^4^ cells/well. After 1, 3, and 5 days incubation in culture medium, the CCK-8 assay was performed to measure cell proliferation on the Gel-DA/DOHA/DMON@Fe hydrogels by a microplate reader (Multiskan MK3, USA) at 450 nm. Furthermore, the Live/Dead staining assay was performed to evaluate cell viability. As regards the immunofluorescent staining, after three days of culture of the cells with the hydrogels, the Gel-DA/DOHA/DMON@Fe hydrogels were permeated in 4% paraformaldehyde and 0.1% Triton X-100. Bovine serum albumin was used to block non-specific binding sites. F-actin filaments were labeled using rhodamine-conjugated phalloidin (Life Technologies) incubated at 37°C in the dark and nuclei were stained with 4′,6-diamidino-2-phenylindole (DAPI, Life). Then, the morphology of the cells that adhered to the hydrogels was observed under a confocal laser scanning microscope (CLSM TCSSP5, Leica, Germany).

#### 2.6.2. Chondrogenic diﬀerentiation of BMSCs induced by hydrogels

The expression of chondrogenic markers, including Col2a1, GAGs, Aggrecan, and SOX 9 was assessed using qRT-PCR. The total RNA was extracted from the BMSCs after culturing for 2 weeks and was reverse-transcribed to cDNA with PrimeScriptTM RT reagent Kit (TaKaRa, Japan) according to the manufacturer’s instructions.

### 2.7. Evaluation of the antioxidative and anti-inﬂammatory ability of the hydrogel in vitro

#### 2.7.1. Intracellular ROS detection

LPS-activated RAW 264.7 murine macrophage cells were incubated with different hydrogels and the ROS-scavenging ability and the corresponding effects on extracellular cytokine expression were evaluated. Briefly, Raw 264.7 cells were seeded on a 12-well plate at a density of 5 × 10^4^ cells per well and incubated overnight at 37°C. Then the cells were activated with LPS (5 μg/mL), followed by the treatment with Gel-DA/DOHA, Gel-DA/DOHA/DMON@Fe, and Gel-DA/DOHA/DMON@Dex@Fe, and incubated for 12 h. The intracellular ROS were detected using DCFH-DA and the labeled ROS were observed under CLSM. The immunofluorescence staining of HIF-1ɑ expression was measured by ﬂow cytometry. Briefly, the cells seeded as same as above at the same density were treated with the different hydrogels fixed with paraformaldehyde (4%, 15 min), collected by trypsinization and analyzed. The intracellular ROS level in the macrophages was also analyzed by ﬂow cytometry, and the RAW 264.7 cell without LPS treatment was considered as the negative group.

#### 2.7.2. Analysis of pro-inﬂammatory cytokines

Similarly, after the incubation, the supernatants were collected and the level of TNF-α and IL-6 was assessed in the culture supernatants by ELISA according to the manufacturer’s protocol. As regards western blot analysis, cells were collected and lysed in cold RIPA buffer for 30 min. Then, the lysates were centrifuged at 12,000 g for 15 min. The obtained total proteins were separated on a polyacrylamide gel, transferred onto a poly(vinylidene difluoride) (PVDF) membrane and blocked with milk. Subsequently, the PVDF membrane was incubated with an anti-COX-2 antibody and incubated at 4°C overnight. The following day, the membrane was immunoblotted with a horseradish peroxidase-conjugated secondary antibody.

### 2.8. Animal surgery and cartilage defect model

The animal studies were performed according to the approval of the Experimental Animal Center of Peking University Shenzhen Hospital. The Sprague Dawley (SD) rats were treated with pentobarbital (3 wt%, 40 mg.kg^−1^). An electric drill was used to create an osteochondral defect of 3.5 mm in diameter and 5 mm in thickness in the right leg of the SD rats through the articular cartilage and subchondral bone of the patellar groove. Next, the rats were randomly divided into four groups (n = 4), such as the control group treated with PBS and the other three groups treated with an intra-articular injection of Gel-DA/DOHA, Gel-DA/DOHA/DMON@Fe, and Gel-DA/DOHA/DMON@Dex@Fe hydrogel. After that, all rats were sacrificed at 8 weeks post-operation, and the knee joint was anatomized and fixed in 4% formaldehyde for 12 h. The harvested samples were stained with hematoxylin and eosin (H&E), toluidine blue, and immunohistochemically COL-II to evaluate the status of cartilage tissue regeneration. Furthermore, immunohistochemical staining of TNF- and IL-6 was performed on related sections for confirming the pro-inﬂammatory cytokine levels after the hydrogels treatment in vivo [24]. All slices were photographed and analyzed using a microscope (IX53, Olympus, Japan).

### 2.9. Statistical analysis

Statistical analysis was performed using two-way *t*-tests between two groups or one-way analysis of variance among multiple groups. The significance level was set as p < 0.05 (*), p < 0.01 (**), and p < 0.001 (***).

## 3. Results and discussion

### 3.1. Synthesis and characterization of DMON@Fe

**Figure 1.**
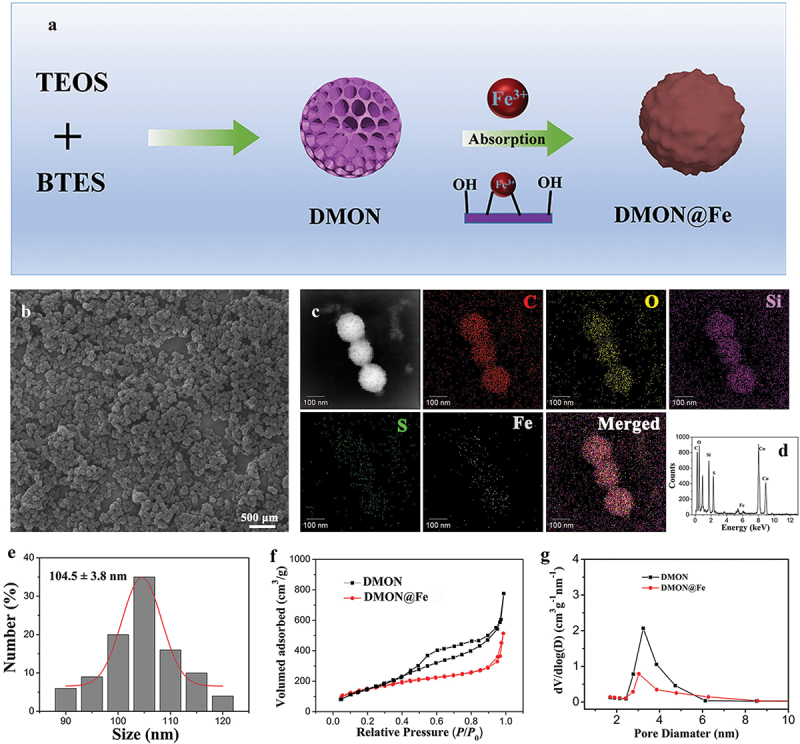
(a) Graphical scheme of the synthesis of DMON@Fe nanoparticles. (b) SEM micrographs of DMON@Fe. (c) Elemental mappings and (d) energy dispersive X-ray (EDX) spectrum of DMON@Fe. (e) Size distribution of DMON@Fe. (f) Nitrogen absorption−desorption isotherm and (g) the corresponding pore-size distribution of DMON and DMON@Fe.

DMON nanoparticles with large radial pore structures and highly accessible surface areas, serving as the robust nanosupports for therapeutic drug delivery, were synthesized following a modification of the Stöber strategy [25,26]. Next, iron doping was performed using DMON as a template through electrostatic interaction between DMON with Fe^3+^, which was continuously deposited on the surface of DMON until the DMON was completely consumed, finally leading to the formation of DMON@Fe (Figure 1(a)). The uniform morphology and spherical shape of DMON@Fe were evident and observed using SEM (Figure 1(b)), the well-formed spherical DMON@Fe had a rough surface. The corresponding average size was approximately 105 nm (Figure 1(e)). The presence of the Fe element on DMON@Fe was demonstrated by the element mapping in the SEM images (Figure 1(c)). According to the EDX analysis, the proportion of Si and Fe elements resulted as being 15.0 wt% and 6.2 wt%, respectively (Figure 1(d)). Additionally, the N_2_ adsorption-desorption isotherms were used to measure the surface and pore size distribution of the as-synthesized DMON and DMON@Fe nanoparticles (Figure 1(f,g)). N_2_ absorption-desorption isotherms of the two nanoparticles could be classified as type IV, conforming to the mesoporous structure [27]. The continuous decrease of the surface (from 315 m^2^•g ^−1^ to 109.6 m^2^•g ^−1^) sequentially follow the Fe absorption. In addition, the pore size of DMON was 3.9 nm, which decreased to 3.1 nm in DMON@Fe, further confirming the successful formation of the Fe-doped DMON nanosystem.

### 3.2. Preparation and characterization of an injectable dual-crosslinking hydrogel

**Figure 2.**
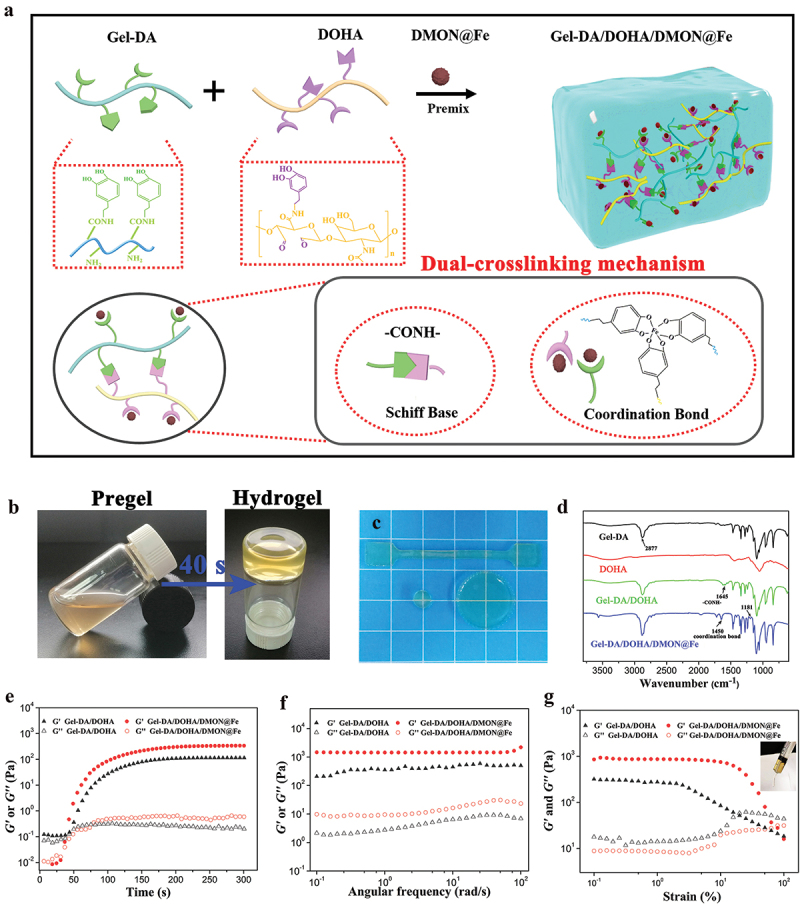
(a) Scheme of Gel-DA/DOHA/DMON@Fe hydrogel synthesis by a dual-crosslinking strategy. (b) Images of the gelation process of the dual-crosslinking hydrogel and (c) the prepared Gel-DA/DOHA/DMON@Fe specimens. (d) the formation of the Gel-DA/DOHA/DMON@Fe hydrogel revealed by FTIR transmittance spectra. The rheological results of the obtained hydrogels: storage modulus (*G*′) and loss modulus (*G*′′) in (e) time sweep, (f) frequency-dependent (at a strain of 1%), and (g) strain-dependent (ω = 6.28 rad/s) oscillatory shear.

In view of the dynamic cartilage injuries, a physicochemical double cross-linked multifunctional hydrogel was proposed (Figure 2(a)). The modified Gel-DA and DOHA were chosen as the main materials of the Gel-DA/DOHA/DMON@Fe hydrogel since they could mimic the ally and structure of the extracellular matrix and could be degraded. The dual-crosslinking hydrogel system was produced by Schiff base bonds between the amino groups in the Gel-DA and the aldehyde groups in the DOHA, and the coordinate bonds between the DMON@Fe and catechol groups in Gel-DA and DOHA. Figure 2(b,c) show that the translucent hydrogel was produced by the mixed pre-gel solution via a simple vortex process. The chemical bond in the hydrogel network was characterized by FTIR spectroscopy (Figure 2(d)). The typical characteristic peak appeared at 1645 cm^−1^ which attributed to imine bonds of the Gel-DA/DOHA and Gel-DA/DOHA/DMON@Fe hydrogel [28], demonstrating the generation of Schiff base bonds based on Gel-DA and DOHA. Besides, Gel-DA/DOHA/DMON@Fe appeared new band at 1181 and 1450 cm ^−1^ respectively compared with Gel-DA/DOHA scaffold [29], which might be the result of the interaction between Fe and Gel-DA/DOHA. These results demonstrated the successful formation of the dual-crosslinking hydrogel based on Schiff base bonds and coordinate bonds between the Fe^3+^ and catechol groups.

Rheological measurements of the gelation process were carried out using time, frequency, and strain sweep to further reveal the gelation mechanism of Gel-DA/DOHA/DMON@Fe hydrogels. The *G*′ value gradually increased from 0 s to 100 s and it intersected the *G*′′ value in the 40th s, revealing the transition point of sol-gelation (Figure 2(e)). The gelatin time of Gel-DA/DOHA/DMON@Fe hydrogel was faster than that of the pure Gel-DA/DOHA sample due to the dual-crosslinking mechanism and possessed a higher equilibrium modulus (*G*_*e*_*′*). The results of the frequency sweep of testing hydrogels are shown in Figure 2(f). The *G′* value remained constant over the whole frequency sweep range, indicating the formation of a well-developed crosslinked network [30].

Besides, despite the strain-dependent oscillatory rheology, the Gel-DA/DOHA/DMON@Fe hydrogels exhibited stress relaxation at strains over 50%, indicating a wide processing range and shear-thinning behavior. A syringe could be used to squeeze the hydrogel without any clogging because of their shear-thinning property, indicating their good injectability (Figure 2(g)). The introduction of DMON@Fe into Gel-DA/DOHA not only formed a coordination bond, but also acted as a physical filler to reinforce the hydrogel.

### 3.3. Mechanical behavior, swelling ratio, and morphology of the hydrogels

**Figure 3.**
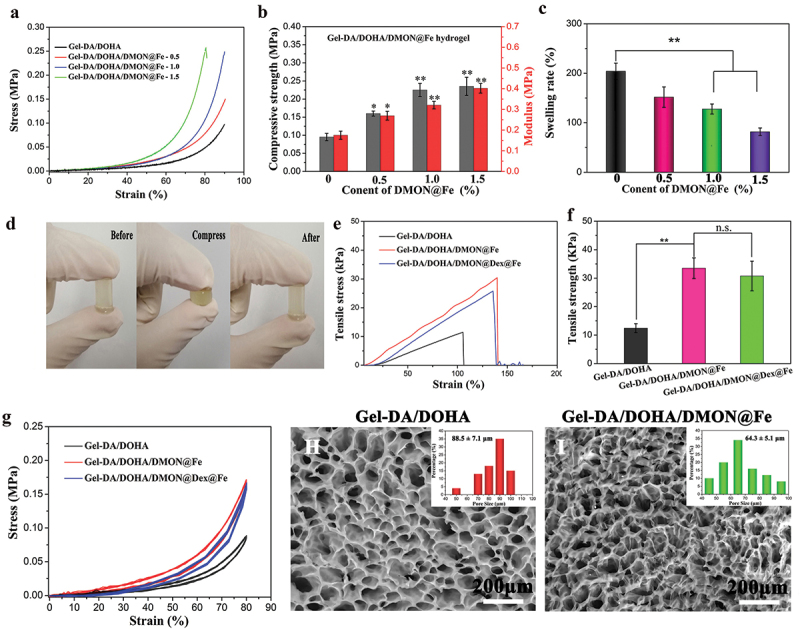
(a) Compressive stress–strain curves of Gel-DA/DOHA/DMON@Fe hydrogels with 0.5, 1.0, and 1.5 wt% DMON@Fe contents and (b) the corresponding results of mechanical strength and Young’s modulus. (c) Equilibrium swelling rate of the hydrogels at 24 h. (d) Images of the compressive behavior for Gel-DA/DOHA/DMON@Fe scaffold. (e) Tensile stress–strain curves of three groups of hydrogels and (f) their corresponding statistical results of tensile strength. (g) Twenty successive compressive loading-unloading cycles for two groups of hydrogels with a strain of 80%. SEM images and statistical pore size of (H) Gel-DA/DOHA and (I) Gel-DA/DOHA/DMON@Fe.

Quantitative mechanical analysis was performed on Gel-DA/DOHA/DMON@Fe scaffold through a typical compressive test. The results of the measurement revealed that the incorporation of DMON@Fe into the Gel-DA/DOHA hydrogel clearly increased its mechanical strength and the excellent mechanical performance was developed by Young’s modulus due to the generation of Fe-catechol coordination bond, as shown in Figure 3(a,b). The compressive strength of the Gel-DA/DOHA/DMON@Fe-1.0 samples significantly increased to 0.23 MPa and showed clear enhancement of mechanical strength compared with neat Gel-DA/DOHA sample (p < 0.01), while the ductility of the hydrogel samples decreased with the addition of 1.5 wt% DMON@Fe. It might be possible that the dual-crosslinking hydrogel prepared under higher DMON@Fe concentration led to weak energy dissipation and lower stress-bearing deformations by aggregation of the nanoparticles [31]. Thus, the Gel-DA/DOHA/DMON@Fe-1.0 was chosen as the optimal component for the subsequent experiments.

The above results were also confirmed by the swelling behavior of these hydrogels. The dual-crosslinking Gel-DA/DOHA/DMON@Fe hydrogels contributed to a smaller swelling rate than the pure Gel-DA/DOHA sample as shown in Figure 3(c). Gel-DA/DOHA/DMON@Fe-1.5 reached a minimum water absorption of 74.6% at 24 h. Similar to the compressive properties, Gel-DA/DOHA/DMON@Fe sample showed a tensile strength of 33.6 kPa, which was 2.6-fold improved over Gel-DA/DOHA hydrogel (Figure 3(e)). Gel-DA/DOHA/DMON@Fe hydrogel did not break when subjected to strong deformation because it formed a dual-crosslinked hydrogel network (Figure 3(d)). It is worth pointing out that the incorporation of anti-inflammatory drugs (Dex) into Gel-DA/DOHA/DMON@Fe network did not apparently influence on the mechanical performance of composite hydrogel system. From the quantitative tensile results (Figure 3(f)), the tensile strength of Gel-DA/DOHA/DMON@Fe with or without Dex hydrogel displayed no significant difference. Next, the 20-cycle loading−unloading compression tests further verified the above conclusion (Figure 3(g)), showing that the formed Gel-DA/DOHA/DMON@Fe or Gel-DA/DOHA/DMON@Dex@Fe hydrogel could withstand 80% of the strain under nearby compressive loading, demonstrating its excellent resilience and toughness properties and promising potential for drug delivery vehicle.

The SEM images of the lyophilized Gel-DA/DOHA and Gel-DA/DOHA/DMON@Fe hydrogels (Figure 3(h,i)) showed similar morphology, whereas Gel-DA/DOHA/DMON@Fe formed a uniform and dense network structure through the coordination of Fe and catechol groups. According to the statistical results analyzed by Image J, the pore size distribution of Gel-DA/DOHA and Gel-DA/DOHA/DMON@Fe hydrogels was 88.5 ± 7.1 and 64.3 ± 5.1 μm, respectively. Obviously, the addition of DMON@Fe resulted in a more denser porous and interconnected microporous three-dimensional architecture owing to the formation of uniform-dense networks via Schiff base bonds and coordinated cross-linking. The interconnected porous structure of Gel-DA/DOHA/DMON@Fe allows the supply of nutrients to the cells, as well can accelerate the chondrogenic differentiation of BMSCs [32].

### 3.4. Construction of Dex controlled release system

**Figure 4.**
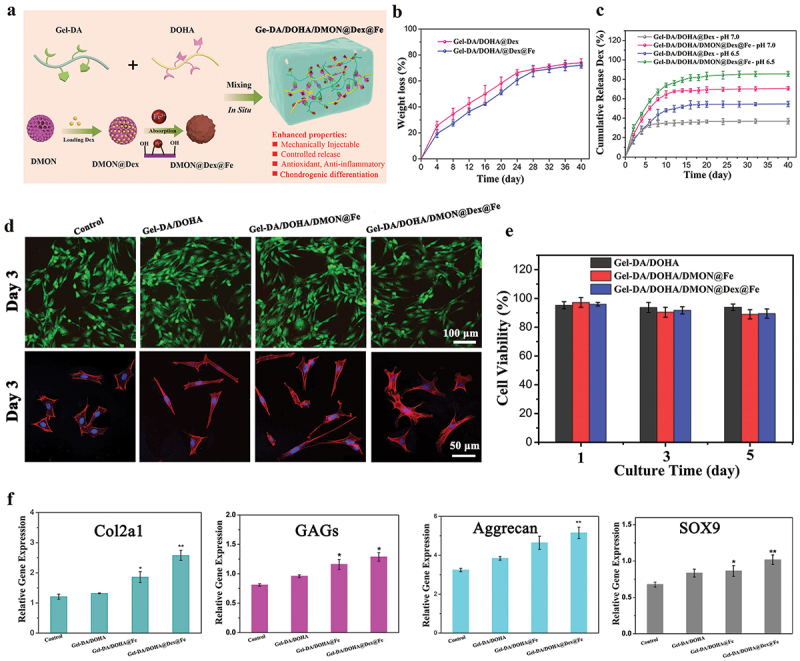
(a) Scheme of Gel-DA/DOHA/DMON@Fe hydrogel in situ drug delivery platform was employed for localized and sustained delivery of Dex. (b) in vitro degradation profiles of the hydrogels in pH 7.4 PBS at 37°C (c) the release curves of Dex from Gel-DA/DOHA and Gel-DA/DOHA/DMON@Fe system in pH 6.5 and 7.0, respectively. (d) Live/Dead staining of BMSCs and CLSM images of BMSCs cultured on different hydrogel scaffolds. (e) CCK-8 assay of the proliferation of BMSCs. (f) RT-qPCR analysis: the gene expression of chondrogenic differentiation of cells after culturing for 14 days.

Glucocorticoids such as Dex are very useful in the treatment of inflammation since they quickly slow the progress of inflammation and relieve the pain [10]. Dex hydrophobic powder was successfully incorporated into DMON using a solution-solvent evaporation method, and was readily absorbed and concentrated into the hydrophobic core of DMON through hydrophobic interactions, and Fe^3+^ was deposited on the surface of the mesoporous shell to prevent the leakage of the entrapped Dex molecules. The obtained DMON@Dex@Fe was mixed with the gel precursor solutions to fabricate Dex-loaded Gel-DA/DOHA/DMON@Fe based on the dual-crosslinking mechanism (Figure 4(a)), and the Gel-DA/DOHA/DMON@Dex@Fe hydrogels showed higher loading efficiency with 92.7 ± 3.9% determined by standard curve (Figure S1), which mainly due to the effect of the denser polymer network structure and the blocking effect between the DMON@Fe and the drug molecules avoids premature release of the drug [33].

The modified Gel-DA and DOHA can be degraded in a physiological microenvironment [29], giving to the Gel-DA/DOHA/DMON@Dex@Fe a preferable biodegradation behavior. Thus, the biodegradation behavior of hydrogels was investigated in PBS solution (pH 7.4). A time-dependent biodegradable behavior was observed, as shown in Figure 4(b). The soaking in PBS for 40 days resulted in a weight loss of the Gel-DA/DOHA@Dex and Gel-DA/DOHA/DMON@Dex@Fe of 73.6 ± 1.1% and 70.4 ± 1.4%, respectively. These degradation results suggested that the addition of DMON@Fe led to a hydrogel system formed by higher crosslink density and a more stable network structure, which is more suitable for the sustained release of Dex and cartilage repair process. The Dex release at pH 6.5 (mimicking the acidic inflammatory microenvironment) was much faster than that at pH 7.0 for both Gel-DA/DOHA and Gel-DA/DOHA/DMON@Fe because of the destruction of the polymer structure at the acidic conditions (Figure 4(c)). Moreover, since the Gel-DA/DOHA/DMON@Fe gel contained Dex, the on-demand drug-releasing pattern was what we expected to observe, realizing a sustained release for over 15 days due to a higher cross-linking density structure of the dual-crosslinking hydrogel. The released amount of Dex from Gel-DA/DOHA/DMON@Fe was more abundant than that released from the Gel-DA/DOHA thanks to the much higher Dex loading content of Gel-DA/DOHA/DMON@Fe than that of Gel-DA/DOHA (2.3 ± 0.4% *vs*. 1.1%).

### 3.5. Cytocompatibility and chondrogenic differentiation assay in vitro

Good cytocompatibility is essential for cartilage tissue engineering [34]. Thus, the cell compatibility of the hydrogels was further estimated. Figure 4(d) shows the Live/Dead staining performed to evaluate the cytotoxicity of the hydrogels, which revealed the presence of a small number of dead cells in the three groups, suggesting that the integration of Dex had no adverse effects on cell growth. In addition, the cell morphology revealed that the BMSCs culture treated with all the hydrogels had a larger diffusion area, and those treated with the Dex-encapsulated Gel-DA/DOHA/DMON@Fe hydrogel still maintained a good slender and fusiform morphology. This was further verified using the CCK-8 kit, which revealed no significant difference in the cell viability between cells treated with the Gel-DA/DOHA, Gel-DA/DOHA/DMON@Fe, and Dex-loaded Gel-DA/DOHA/DMON@Fe group over 3 days (Figure 4(e)). Almost all cells in the experimental groups still maintained the viability of over 85% after 5 days of culture with the hydrogels, which was consistent with the Live-Dead assay results. All these results indicated that these hydrogels possessed good cytocompatibility and might serve as an effective matrix to support the adhesion and growth of BMSCs.

Previous studies have reported that silica-based nanoparticles play an important role in the process of cartilage synthesis and the integrity of the extracellular matrix [29,35]. The chondrogenic differentiation genes (Col2a1, GAGs, aggrecan, and SOX9) were examined for further investigating the effect of dual-crosslinking hydrogels on chondrogenic differentiation capacity. Compared with the control group, the effect on cell chondrogenesis was significantly enhanced in Gel-DA/DOHA/DMON@Fe and Gel-DA/DOHA/DMON@Dex@Fe (Figure 4(f)). Collectively, these results demonstrated that the chondrogenic differentiation of BMSCs could be effectively promoted by Gel-DA/DOHA/DMON@Fe, being superior to that of Gel-DA/DOHA, revealing a promising strategy for the cartilage repair.

### 3.6. Antioxidative and anti-inﬂammatory performance in vitro

**Figure 5.**
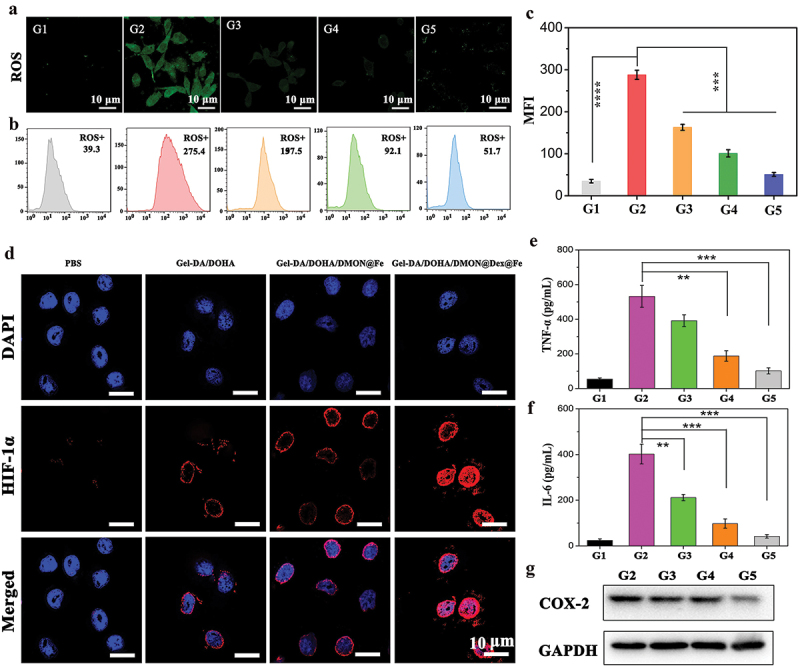
(a) ROS levels in Raw 264.7 cells visualized with a confocal laser scanning microscope (CLSM). G1: PBS, G2: LPS (positive control), G3: LPS + Gel-DA/DOHA, G4: LPS + Gel-DA/DOHA/DMON@Fe, G5: LPS + Gel-DA/DOHA/DMON@Dex@Fe. (b) Corresponding ROS levels in Raw 264.7 cells were analyzed by flow cytometry and (c) their quantitative analysis of the mean fluorescence intensities (MFI). (d) the evaluation of hypoxia in Raw 264.7 cells treated with PBS, Gel-DA/DOHA, Gel-DA/DOHA/DMON@Fe and Gel-DA/DOHA/DMON@Dex@Fe by staining with HIF-1α (red). Results of the expression of the inﬂammatory factors by ELISA kit. (e) TNF-α and (f) IL-6. (G) COX-2 expression in Raw 264.7 cells after various treatments using Western blot.

The excessive production of ROS leads to inflammation [36,37]. Therefore, the ROS level of the activated RAW264.7 cells treated with the three groups of hydrogels was examined through ﬂuorescence microscopic imaging (Figure 5(a)) and ﬂow cytometry assay (Figure 5(b)). ROS were overproduced after LPS stimulation, resulting in a bright green signal in Raw 264.7 cells. Gel-DA/DOHA, Gel-DA/DOHA/DMON@Fe, and Gel-DA/DOHA/DMON@Dex@Fe effectively decreased the ROS levels in the activated Raw 264.7 cells. This result was confirmed by the quantitative ﬂow cytometry assay, which revealed that the ﬂuorescence intensity was dramatically decreased after the treatment with Gel-DA/DOHA/DMON@Fe and Dex-loaded Gel-DA/DOHA/DMON@Fe gel, indicating an efficient ROS depletion. On the one hand, catechol-containing hydrogels have good antioxidative ability leading to a significant decrease in ROS [38]. On the other hand, the DMON@Fe-coordination between catechol groups with the redox cycling reaction between the Fe^2+^/Fe^3+^ could also remove some ROS, and the sustained release of Dex, in turn, suppressed the inflammatory response, resulting in less production of ROS [39,40]. Quantitative ﬂow cytometry results further confirmed these findings (Figure 5(c)). The ﬂuorescence intensities of G2 group exhibited higher level than other three group treated by various hydrogels (***P < 0.001), especially, the Dex-loaded Gel-DA/DOHA/DMON@Fe group possess the optimal ROS scavenging ability, which is consistent with the confocal results. The above results indicated that the introduction of the catechol groups and DMON@Fe nanoparticles conferred excellent antioxidant activity to the hydrogels.

Inspired by the ROS scavenging activity and the on-demand drug releasing ability of the Gel-DA/DOHA/DMON@Dex@Fe, hypoxia was assessed by the evaluation of HIF-1α (red staining). Both the Gel-DA/DOHA/DMON@Fe and Dex-loaded Gel-DA/DOHA/DMON@Fe resulted in a higher expression of HIF-1α compared to the PBS treated group, confirming the efficient removal of ROS as well (Figure 5(d)), being the key factor promoting the cartilage development. The presence of HIF-1α not only promote the expression of SOX9, which is a key regulator of chondrogenic differentiation, but it also switches the metabolism from glucose oxidation to glycolysis to fulfill the energy requirements that regulate the proliferation and survival of chondrocytes [39].

Moreover, the expression of the inﬂammatory factors TNF-α and IL-6 was also assessed by ELISA Kit. Their downregulation was observed in the Gel-DA/DOHA/DMON@Fe and Gel-DA/DOHA/DMON@Dex@Fe group, which resulted in a significant downregulation of TNF-α and IL-6 (Figure 5(e,f)). The inhibition of COX-2 expression by the hydrogels was also evaluated by western blot (Figure 5(g)). The Dex-loaded Gel-DA/DOHA/DMON@Fe group showed the best effect in inhibiting the expression of inflammatory COX-2 compared to the LPS-activated macrophage cells used as a control group that displayed an apparent COX-2 expression. These results were in good agreement with the ROS results, demonstrating that the Dex delivery mediated a better inhibition of the TNF-α and IL-6 expression, and the combined antioxidative and anti-inﬂammatory effect might lead to the most significant inhibition in the progression of inflammation.

### 3.7. Cartilage regeneration assessment of Gel-DA/DOHA/DMON@Dex@Fe hydrogel

**Figure 6.**
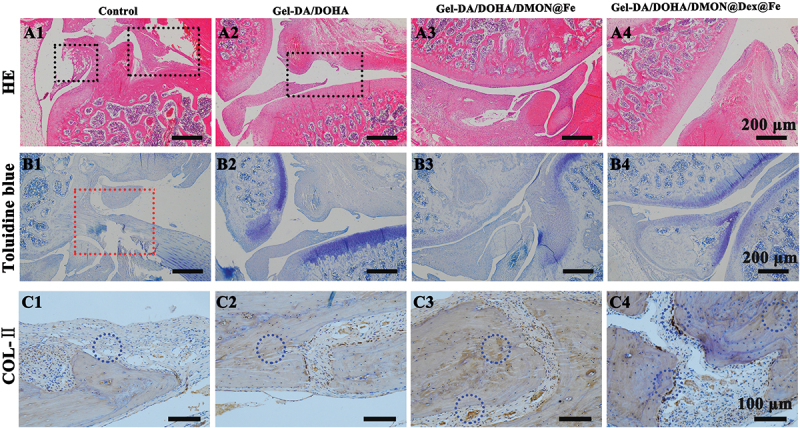
(a–c) Representative (a) H&E staining, black frame indicate cartilage; (b) Toluidine blue staining, red frame indicate irregular regenerated tissues; (c) COL-II immunohistochemical staining of the cartilage sections in different groups after 8 weeks of treatment, blue circle indicate the expression of COL-ⅱ(dark brown represents its high expression).

Based on the promising chondrogenic differentiation, as well as the antioxidative and anti-inflammatory activity of Gel-DA/DOHA/DMON@Dex@Fe *in vitro*, a rat model was used to evaluate its therapeutic effect on cartilage injury. The defects treated with Gel-DA/DOHA/DMON@Fe hydrogel were filled with more uniform and better cartilage, as observed by HE staining compared with the untreated defect (black frame) (Figure 6(a)). Similarly, the groups treated with Dex-loaded Gel-DA/DOHA/DMON@Fe hydrogel had relatively smoother repaired cartilage surfaces, the new cartilage tissue was more mature and the boundary of the repaired cartilage was more complete compared with the empty Gel-DA/DOHA group, indicating that the combination of Gel-DA/DOHA/DMON@Fe and Dex was more effective in the treatment of OA. Similarly, the Toluidine blue staining indicated that the regenerated proteoglycan (shown in blue) regenerated the hyaline cartilage in Gel-DA/DOHA/DMON@Fe and Dex-loaded Gel-DA/DOHA/DMON@Fe (Figure 6(b)). In contrast, irregular regenerated tissues were found in the untreated group. These results were consistent with the findings of our further study on collagen II (chondrogenic markers) stained by immunohistochemistry. Collagen II staining was widespread in the Gel-DA/DOHA/DMON@Fe and Dex-loaded Gel-DA/DOHA/DMON@Fe. The staining color was uniformly distributed among the layers of the articular cartilage. The expression of collagen II was significantly increased (dark brown) compared with its expression in the control group (Figure 6(c)). This result further proved the superior efficacy of the Gel-DA/DOHA/DMON@Dex@Fe scaffold for the regeneration and remodeling of the cartilage defect in rats.

### 3.8. Anti-Inflammatory effect of hydrogels in vivo

**Figure 7.**
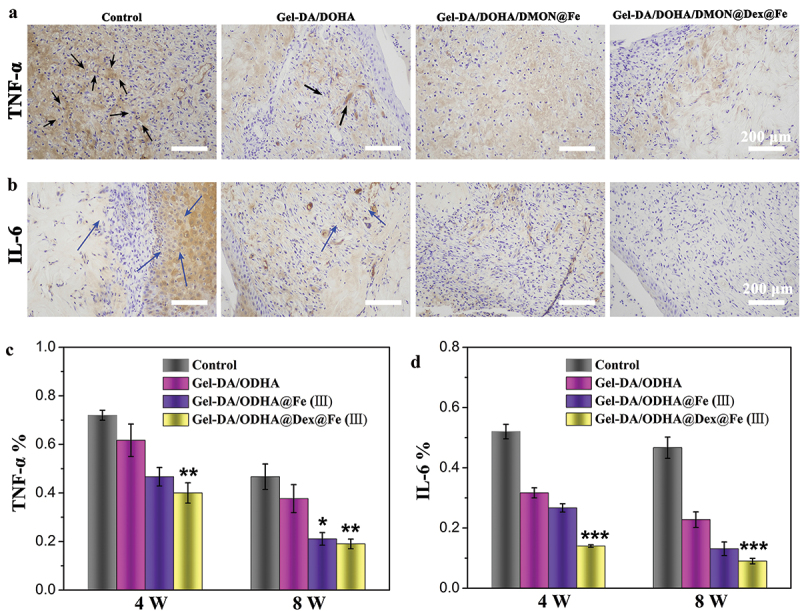
Immunohistochemical staining of pro-inﬂammatory factors (a) TNF-α, (b) IL-6 after different treatment for 8 weeks (black and blue arrow represent the expression of TNF-α and IL-6 respectively); (c, d) its corresponding results of semi-quantitative analysis of immunohistochemical staining.

As previously reported, pro-inﬂammatory cytokines are highly expressed in cartilage defects, thus triggering the development of OA [41,42]. Therefore, the expression of pro-inflammatory factors, such as TNF-α and IL-6 (brown color), was further analyzed by immunohistochemical staining after the treatment of different samples. Figure 7(a–d) shows that the expression of these inﬂammatory factors in the Gel-DA/DOHA group was lower than that in the first group (control group). In particular, the Gel-DA/DOHA/DMON@Fe and Dex-loaded Gel-DA/DOHA/DMON@Fe group exerted an evident down-regulation of these pro-inﬂammatory factors compared with another two groups, where the expression of TNF-α and IL-6 was the lowest after the treatment of Gel-DA/DOHA/DMON@Dex@Fe, all results consistent with the *in vitro* results. Notably, the combined treatment of the cartilage defect with Gel-DA/DOHA/DMON@Dex@Fe efficiently scavenged the excess of ROS to facilitate cartilage regeneration and maintain lower levels of the pro-inﬂammatory cytokine, showing its superior anti-inﬂammatory performance compared with any single treatment.

## 4. Conclusion

In conclusion, an injectable multifunctional hydrogel was developed by Schiff base bonds between amino groups in Gel-DA and the aldehyde groups in DOHA, and coordinate bonds between the DMON@Fe and catechol groups in Gel-DA and DOHA. The mechanical properties of the hydrogel scaffolds could be effectively enhanced by the incorporation of DMON@Fe nanoparticles with an appropriate porosity and fast gelation performance. Furthermore, the resulting dual-crosslinking hydrogels showed a favorable degradation rate, good cytocompatibility, and realized a sustained release of Dex for 15 days. More importantly, the Gel-DA/DOHA/DMON@Dex@Fe system showed an effective chondrogenic differentiation ability by inducing the activation of HIF-1ɑ, leading to a hypoxic microenvironment and significant down-regulation of inflammatory response. The *in vivo* study demonstrated that the dual-crosslinking hydrogel enhanced the efficacy of the treatment on cartilage defects by the effective removal of the ROS and the inhibition of TNF-α, IL-6. Thus, a novel innovative strategy could be provided by such multifunctional system for long-term articular cartilage repair, as a promising candidate for protection against perifocal OA.

## Supplementary Material

Supplemental Material
