# Supplementary material for: Multifunctional injectable hydrogel for effective promotion of cartilage regeneration and protection against osteoarthritis: combined chondroinductive, antioxidative and anti-inﬂammatory strategy
Source: Sci Technol Adv Mater. 2022 Jun 1;23(1):361–75. doi: 10.1080/14686996.2022.2076568 (PMC9176362; doi:10.1080/14686996.2022.2076568)
Supplement: Supplemental Material [file TSTA_A_2076568_SM8844.docx]

**Supporting information**

**Multifunctional injectable hydrogel for effective promotion of cartilage regeneration and protection against osteoarthritis: combined chondroinductive,** **antioxidative and anti-inﬂammatory strategy**

Xueping Dong^1, 2^, Canfeng Li^1^, Mengdi Zhang^1^, YiKun Zhao^1^, Zhen Zhao^1^, Wenqiang Li^3, *^, Xintao Zhang^1, *^

^1^Department of Sports Medicine and Rehabilitation, Peking University Shenzhen Hospital, Shenzhen 518036, PR China

^2^School of Clinical Medicine, Weifang Medical University, Weifang 261000, PR China

^3^Guangzhou Sport University, Guangzhou 510500, PR China

^*^Corresponding authors: Wenqiang Li (gztylwq@foxmail.com); and Xintao Zhang [(zhangxintao@sina.com)](mailto:(zhangxintao@sina.com))

**1.1 Dex standard calibration curve**

The prepared Dex solutions of 5 known concentrations, i.e. 30, 20, 10, 5, 2.5 μg/mL were measured by a UV-Vis spectrophotometry at the wavelength of 242 nm to set the standard curve.

Figure S1. The Dex standard calibration curve
